# A New View of Genome Organization Through RNA Directed Interactions

**DOI:** 10.3389/fcell.2020.00517

**Published:** 2020-07-14

**Authors:** Gabriel Khelifi, Samer M. I. Hussein

**Affiliations:** ^1^Department of Molecular Biology, Medical Biochemistry and Pathology, Université Laval, Québec, QC, Canada; ^2^Université Laval Cancer Research Center, Université Laval, Québec, QC, Canada; ^3^Oncology Division, Centre Hospitalier Universitaire (CHU) de Québec-Université Laval Research Center, Québec, QC, Canada

**Keywords:** RNA-DNA interactions, long non-coding RNAs, chromatin modifying complex, chromatin structure, genome organization, RNA-RNA interactions

## Introduction

**Figure 1:**
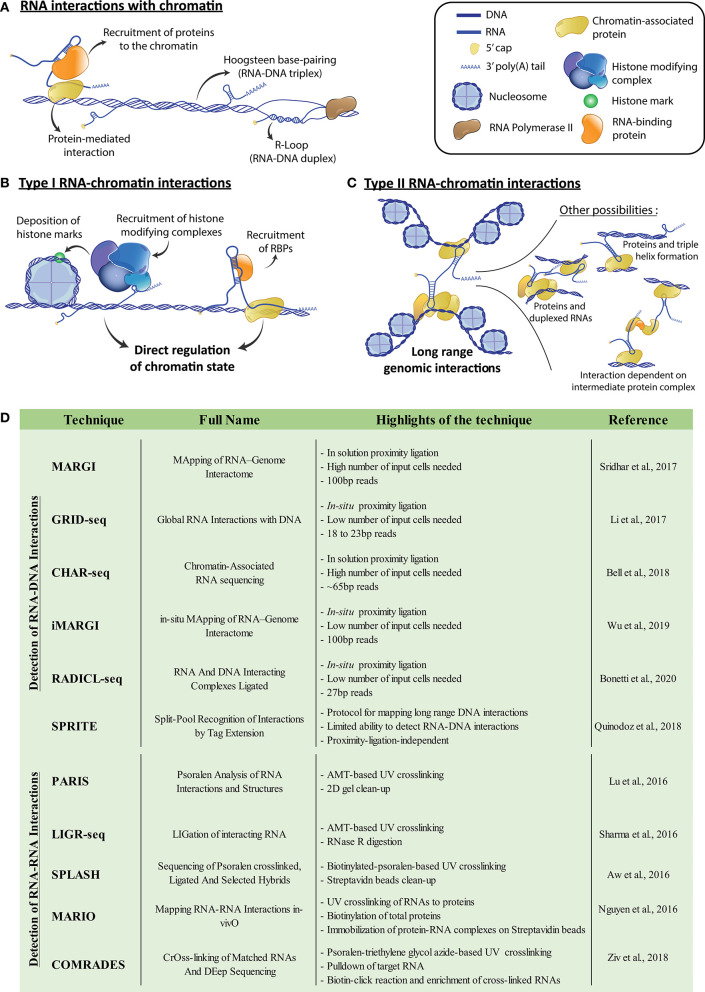
**(A)** Potential means of chromatin-RNA association. Interactions can be mediated by protein intermediates or rely on either Hoogsteen base-pairing to form RNA-DNA triplexes, or Watson-Crick base-pairing to form RNA-DNA duplexes such as R-loops. **(B)** Representation of potential type I RNA-chromatin interactions, where RNAs recruit associated proteins to the chromatin, enabling the modulation of genomic accessibility. For example, recruited proteins can be part of chromatin modifying complexes or act as transcription factors. **(C)** Representation of potential type II RNA-chromatin interactions, where RNAs interact with genetically distant loci and modulate genome architecture, potentially bringing them physically closer. Interactions can be direct associations of the RNA with the DNA, or be mediated either by chromatin-bound proteins, or RNA duplexing. RNA bound protein complex intermediates can also generate indirect interactions. **(D)** Table of referenced techniques for identification of genome and transcriptome-wide DNA-RNA or RNA-RNA interactions, respectively. For more details, we refer the reader to the corresponding publications within the table. AMT, 4′-aminomethyl-4,5′,8-trimethylpsoralen.

Over the past few decades, we have come to appreciate the complexity of processes regulating chromatin architecture. Ranging from chromatin accessibility (Klemm et al., 2019) to long-range genome organization (Cremer and Cremer, 2001), a wide array of mechanisms is used by the cell to control gene expression. We have recently begun to understand the primordial role of non-coding RNAs (ncRNAs) in such processes and can now declare that several types of RNAs are essential to the regulation of gene expression (Cech and Steitz, 2014). In the nucleus, long non-coding RNAs (lncRNAs) (Bonasio and Shiekhattar, 2014), together with enhancer RNAs (eRNAs) (Li et al., 2016a), stable intronic sequence RNAs (sisRNAs) (Chan and Pek, 2019) and various other classes of transcripts (reviewed in (Li and Fu, 2019)) come together to ensure tight regulation of the chromatin. LncRNAs represent transcripts of more than 200 nucleotides that do not contain any apparent open reading frame (Marchese et al., 2017). While some lncRNAs are localized and active in the cytosol (Noh et al., 2018), many are nuclear and implicated in transcriptional regulation (Vance and Ponting, 2014). These nuclear lncRNAs can modulate the expression of genes through interactions with DNA or chromatin-associated proteins (Bonasio and Shiekhattar, 2014; Figure 1A). eRNAs are abundantly transcribed RNAs generated from enhancer regions (Li et al., 2016a). They modulate enhancer activity potentially through interactions with the mediator complex, transcription factors or chromosomal looping factors. Lastly, sisRNAs represent RNAs containing intronic sequences, and increasing evidence shows that several of them act on chromatin regulation (Chan and Pek, 2019). All of these various classes of chromatin-associated RNAs are essential to the regulation of gene expression (Li and Fu, 2019). Some transcripts appear to be “*cis*-acting,” influencing the expression of genes within their own chromosome, while others control transcriptional processes on other chromosomes in *trans*. Some *cis*-acting RNAs have been shown to function through the formation of R-loops with the complementary sequence from their transcribed loci and affect local gene expression, as is the case with *GATA3-AS1* and *VIM-AS1* (Boque-Sastre et al., 2015; Gibbons et al., 2018). Both *cis*- and *trans*-acting RNAs can affect gene expression through direct RNA-DNA contacts (i.e., RNA-DNA triplexes), as employed by *MEG3* and *KHPS1* (Mondal et al., 2015; Blank-Giwojna et al., 2019), or indirectly through protein intermediates such as the interaction of *FIRRE* lncRNA with the SAF-A protein (Hacisuleyman et al., 2014; Figure 1A). These interactions often require specific RNA “domains,” such as motifs recognized by chromatin bound proteins, or RNA-DNA triplex-forming motifs such as polypurine tracts (Li et al., 2016b; Li and Fu, 2019). RNAs exhibiting interactions in both *cis* and *trans* have also been described, for example: the lncRNAs *FIRRE* (Hacisuleyman et al., 2014; Lewandowski et al., 2019) and *ANRIL* (Kong et al., 2018). This hints at the great complexity of processes regulated by chromatin-associated RNAs.

We postulate that RNAs acting on chromatin can be functionally separated in two groups, following how they affect the transcriptional landscape of the cell. First, RNAs can act locally to where they bind (i.e., short-range) on the structure of the chromatin itself, for example, by modifying its accessibility through the recruitment of structural protein factors or protein complexes that establish chromatin marks (Type I) (Figure 1B). Various Type I RNAs have been characterized. For example, the lncRNAs *HOTAIR* and *FENDERR* and the sisRNA generated from intronic sequences of *SMYD3*, do so in part by recruiting the PRC2 repressive complex to their genomic binding sites (Guil et al., 2012; Grote et al., 2013; Mozdarani et al., 2020). Second, RNAs can control the organization of the genome, which we define as Type II interactions. They do so by promoting long-range chromatin interactions and the bridging of distant genomic loci (Figure 1C). Type II RNAs include examples such as the lncRNAs *LUNAR1* or *Kancr*, both involved in chromosomal looping and activation of genes near the loop anchor points (Trimarchi et al., 2014; Li et al., 2020). Additionally, some lncRNAs can potentially exhibit both types of interactions. For example, the lncRNA *XIST* is transcribed from the X-chromosome and is directly implicated in its inactivation (XCI) (Loda and Heard, 2019). During this process, *XIST* transcripts recruit protein factors necessary for XCI, highlighting a Type I mechanism (Chu et al., 2015). Upon XCI, progressive compaction of the X-chromosome occurs and allows *XIST* to spread on the whole X-chromosome, which results in global heterochromatinization (Engreitz et al., 2013). Moreover, the multiple domains which enable *XIST* to attach to X-chromosome-bound proteins and the *XIST*-dependent X-chromosome conformation, hint at a potential Type II mechanism for this lncRNA in XCI.

While a growing number of RNAs acting on gene expression are being characterized, we still lack a bigger picture on how prevalent these interactions are, and on the importance of the proposed mechanisms. The first major obstacle in delineating which RNAs act on chromatin regulation resides in the immense number of existing transcripts. For example, estimates place the number of potentially functional lncRNAs in the tens of thousands (Marchese et al., 2017), and characterization of functional eRNAs or sisRNAs is still too early to grant accurate estimates (Li et al., 2016a; Chan and Pek, 2019). A tremendous amount of work lies ahead to fully understand the above-mentioned processes. Understanding where and how RNAs interact with chromatin, and the resulting effect on gene regulation, therefore remains an upcoming challenge in the characterization of the global transcriptional landscape.

## Genome-Wide Cataloging of RNA-Chromatin Interactions

The past 20 years have been marked by the development of several techniques aiming to map long-range chromatin interactions and to decipher genome architecture. Efficient techniques now include direct ligation of proximal DNA fragments (3C) (Kempfer and Pombo, 2020), ligation of barcodes to interacting DNA fragments (SPRITE) (Quinodoz et al., 2018), or physical isolation of thin nuclear sections and analysis of the DNA contained within (GAM) (Beagrie et al., 2017). The 3C-based methodologies have inspired new protocols to resolve RNA-DNA interactions, such as MARGI (Sridhar et al., 2017), GRID-seq (Li et al., 2017), CHAR-seq (Bell et al., 2018), iMARGI (Wu et al., 2019), and RADICL-seq (Bonetti et al., 2020). Moreover, SPRITE was also adapted to reveal RNA-DNA interactions (Quinodoz et al., 2018; Figure 1D). Therefore, a vast number of datasets showing RNA-chromatin interactions now exists for diverse cellular contexts. MARGI has already enabled the identification of Type I mechanisms implicating RNA-chromatin interactions in the establishment of various chromatin marks (Sridhar et al., 2017). Additionally, GRID-seq showed that an enrichment of RNA-chromatin interactions is implicated in the role of super-enhancers (Li et al., 2017), representing potential Types I and II RNA-chromatin interactions. GRID-seq was also used to detect the diverse genomic binding sites of *XIST*, both locally to its transcription site, but also throughout the X chromosome. It also highlighted the sites of initiation of XCI via *XIST*. These findings establish the major importance of such high-throughput techniques in characterizing different mechanisms responsible for the tight control of chromatin by RNAs.

## Limitations of Current Methodologies For PROBING RNA-Chromatin Interactions

However, limitations are present in the developed techniques, and many challenges still lie ahead to fully understand how chromatin is regulated by RNAs. First, the developed techniques generally exhibit a high prevalence for RNA reads that map to introns, revealing a widespread capture of nascent RNAs from loci undergoing transcription (actively transcribed RNAs) (Li et al., 2017; Bonetti et al., 2020). During their transcription process, RNAs can be captured via fixation to their genomic loci, therefore appearing as mapped interactions. These highly abundant interactions represent a major contaminant of such experiments, potentially overshadowing actual functional interactions. Their presence should be accounted for to prevent them from affecting the sequencing depth achieved for functional interactions and to allow for the detection of lower-abundance RNA-chromatin interactions. To reduce the effect of nascent RNA-bias, RADICL-seq uses a controlled RNase H digestion step (Bonetti et al., 2020). Although this step does indeed reduce this bias, a significant portion of nascent RNA still remains, and it would be necessary to explore additional ways to remove these unwanted RNAs.

Another main limitation in the current protocols and available datasets resides in the relatively small size of the sequenced tags corresponding to the DNA and its interacting RNA (Li et al., 2017; Sridhar et al., 2017; Bell et al., 2018; Quinodoz et al., 2018; Wu et al., 2019; Bonetti et al., 2020; Figure 1D). Indeed, their short size results in poor mapping of the obtained DNA-RNA pairs to the genome and transcriptome. This problem is further amplified when working with transcripts containing repeated sequences. For example, lncRNAs generally possess a large number of transposable elements (TEs) and other repeated sequences (Johnson and Guigó, 2014), and 83% of lncRNAs contain one or more TEs, compared to only 6% of mRNAs (Kelley and Rinn, 2012). Interestingly, some TEs are implicated in localization of RNA transcripts to the nucleus (Lubelsky and Ulitsky, 2018), which further highlights a role in genome regulation. These TEs have also emerged as important domains in lncRNA function, with several cases demonstrating that an embedded TE acts as the RNA's functional motif (Johnson and Guigó, 2014). These TEs can enable interactions with complementary sequences in DNA or other RNAs, as seems to be the case with the lncRNAs *ANRIL* (Holdt et al., 2013) and *LEADeR* (Profumo et al., 2019). Therefore, a loss in reads corresponding to repeat elements due to an inability to map them represents a major potential hurdle in the current protocols. This is evident in the relatively low percentage of uniquely mapped reads for protocols such as GRID-seq or RADICL-seq (14 and 45%, respectively) (Li et al., 2017; Bonetti et al., 2020). The higher read mapping observed with RADICL-seq compared to GRID-seq is due to an additional 7 base-pairs in the final read length (Bonetti et al., 2020). These protocols use restriction enzymes to generate sequence lengths of around 20 base-pairs for GRID-seq and 27 for RADICL-seq for both RNA and DNA tags (Figure 1D). The increase in read length with RADICL-seq allows mapping of some reads corresponding to TE-containing RNAs. This enabled the authors to determine that transcripts containing TEs are indeed differentially engaged in interactions with chromatin, once again hinting at the importance of TEs in chromatin regulation. Other methodologies, namely MARGI (Sridhar et al., 2017) and iMARGI (Wu et al., 2019), circumvent this limitation through a protocol that preserves the full length of the respective RNA and DNA tags. This enables the generation of libraries containing longer fragments and results in higher mapping of reads.

Thus far, these techniques have relied on “short-read” sequencing technologies, which do not fully overcome the challenge of mapping repeated and complex sequences within RNAs (Dijk et al., 2018). To remediate this problem, one option would be to incorporate “long-read” sequencing technologies to these protocols. Indeed, throughout the last 10 years, the rise in the availability of such sequencing techniques has meant that more and more laboratories can get access to this technology. While it is still in its early stages compared to “short-read” sequencing approaches, the sequencing of long DNA or RNA fragments, ranging from a few hundred nucleotides to tens of kilobases, results in a more accurate alignment of repeat sequences (Dijk et al., 2018). A higher percentage of DNA and RNA pairs should therefore be uniquely mapped, even in the presence of interactions dependent on the complementarity of repeat elements. Additionally, long-read sequencing could provide more detailed information on the RNAs interacting with chromatin. For one, the specific isoforms of RNAs which interact with chromatin could be detected. Also, detection of nascent RNAs would be more precise, as current analysis only take intronic reads into account when counting for nascent RNAs, whereas long-read sequencing will reveal the whole transcript. Therefore, long-read sequencing represents a very promising new tool for further iterations of such protocols.

## RNA-RNA Interactions to Help Understand LNCRNA-Chromatin Interactions

While it is clear now that chromatin-associated RNAs affect the structure and regulation of chromatin, the role of RNA-RNA interactions (RRIs) is not as well-explored in this context. Due to RNA's inherent ability to base pair and form complex higher order structures, it can simultaneously interact with DNA and multiple RNA and protein molecules (Lu and Chang, 2018). Inter-RNA interactions could enable Type II interactions, through base-pairing between two chromatin-associated RNAs (Figure 1C). Meanwhile, intra-RNA interactions, through the RNA's secondary structure, may help in identifying sites available for binding to chromatin or RNA-binding proteins. With these features in mind, we expect that the associations revealed by the techniques probing RNA-chromatin interactions will serve as starting points for integration of RRI networks involved in chromatin regulation. Various protocols recently aimed to investigate RRIs on a transcriptome-wide scale, such as PARIS (Lu et al., 2016), LIGR-seq (Sharma et al., 2016), SPLASH (Aw et al., 2016), and MARIO (Nguyen et al., 2016). Additionally, another technique, COMRADES (Ziv et al., 2018), initially intended to probe RRIs for a single RNA, could also be used for genome-wide RRIs (Figure 1D). These protocols crosslink RNA duplexes, to reveal both RRIs and, to some extent, the secondary structure of every RNA. However, the limitations highlighted for RNA-DNA probing techniques, such as read length and unique mapping of repeated sequences, still apply to RRI-probing protocols. These techniques have nevertheless proven to be instrumental in revealing several cellular processes dependent on RRIs. For example, PARIS highlighted structural folding patterns in the *XIST* A-repeats, which is necessary for binding of SPEN (Lu et al., 2016), a transcriptional repressor involved in X-inactivation (Chu et al., 2015). Analysis of RRIs in combination with RNA localization of *XIST* on the chromatin by GRID-seq (Li et al., 2017) consequently highlights the potential Type I and II mechanisms of *XIST* in XCI. This example demonstrates how combining RRI data to an integrative map of the genome organization extracted from 3C or other related techniques, coupled with existing RNA-chromatin interaction information, will provide a better understanding of the complex mechanisms behind chromatin regulation. Long-range, indirect chromatin interactions mediated by several duplexed RNAs, or by protein complexes exhibiting RNA-binding functions (Figure 1C) will only then become more apparent. Overall, these types of studies provide a more complete view on the complexity of genome organization and chromatin structure.

## Concluding Remarks

Taken together, the methods described here represent useful tools for elucidating the role of RNA-DNA and RNA-RNA interactions in gene expression regulation. While various improvements are still needed, the existing datasets represent a comprehensive look of how a genome could be organized through RNA interactions. Several RNA-DNA interactions have now been cataloged either through these genome-wide techniques or through specific RNA directed techniques. Of these, lncRNAs seem to represent an important fraction of the factors that regulate gene expression, chromatin accessibility and genome organization. In addition, these RNAs are not limited only to chromatin binding but may act as conduits to bring in other types of interactors, such as other RNAs, RNA-binding proteins, and transcriptional complexes. All these elements combined together help forge the transcriptional landscape necessary to maintain and transition between defined cell states.

## Author Contributions

GK and SH conceived and wrote the manuscript. All authors contributed to the article and approved the submitted version.

## Conflict of Interest

The authors declare that the research was conducted in the absence of any commercial or financial relationships that could be construed as a potential conflict of interest. The handling editor declared a shared affiliation with the authors though no other collaboration at time of review.
